# Enhancing size based size separation through vertical focus microfluidics using secondary flow in a ridged microchannel

**DOI:** 10.1038/s41598-017-17388-w

**Published:** 2017-12-12

**Authors:** Bushra Tasadduq, Wilbur Lam, Alexander Alexeev, A. Fatih Sarioglu, Todd Sulchek

**Affiliations:** 1 School of Electrical and Computer Engineering, Georgia Institute of Technology, Atlanta, GA USA; 2 NED University of Engineering & Technology, Karachi, Pakistan; 3 Coulter Department of Biomedical Engineering, Georgia Institute of Technology, Atlanta, GA USA; 4 Woodruff School of Mechanical Engineering, Georgia Institute of Technology, Atlanta, GA USA

## Abstract

High throughput size based separation and sorting of bioparticles and cells is critical to a variety of biomedical processing steps for medical diagnostics and pharmaceutical purification. Improving microfluidic size-based particle/cell sorting is a challenge to better address the need for generating more homogeneous subpopulations for study and use. We propose a novel advance to microfluidic sorting devices that uses three-dimensional focusing of the sample to optimally position particles to amplify the size-dependent differences in trajectories caused by differential secondary flows. The result is an increase in the purity of small particles by 35- fold and large particles by 8-fold in comparison to unfocused flow. Our simulated and experimental data reveal for the first time that positioning particles in three-dimensional space can be used to better leverage the differential lateral movement of particles with different sizes as they flow in microchannel with transverse secondary flows. The focusing approach may also be useful to improve positioning of particles with inertial channels with multiple equilibrium positions. This technique performs continuous-flow, high throughput size based sorting of millions of particles and cells in a minute without any pre and post-processing. We have also demonstrated improved enrichment and recovery of white blood cells from human blood.

## Introduction

**Figure 1:**
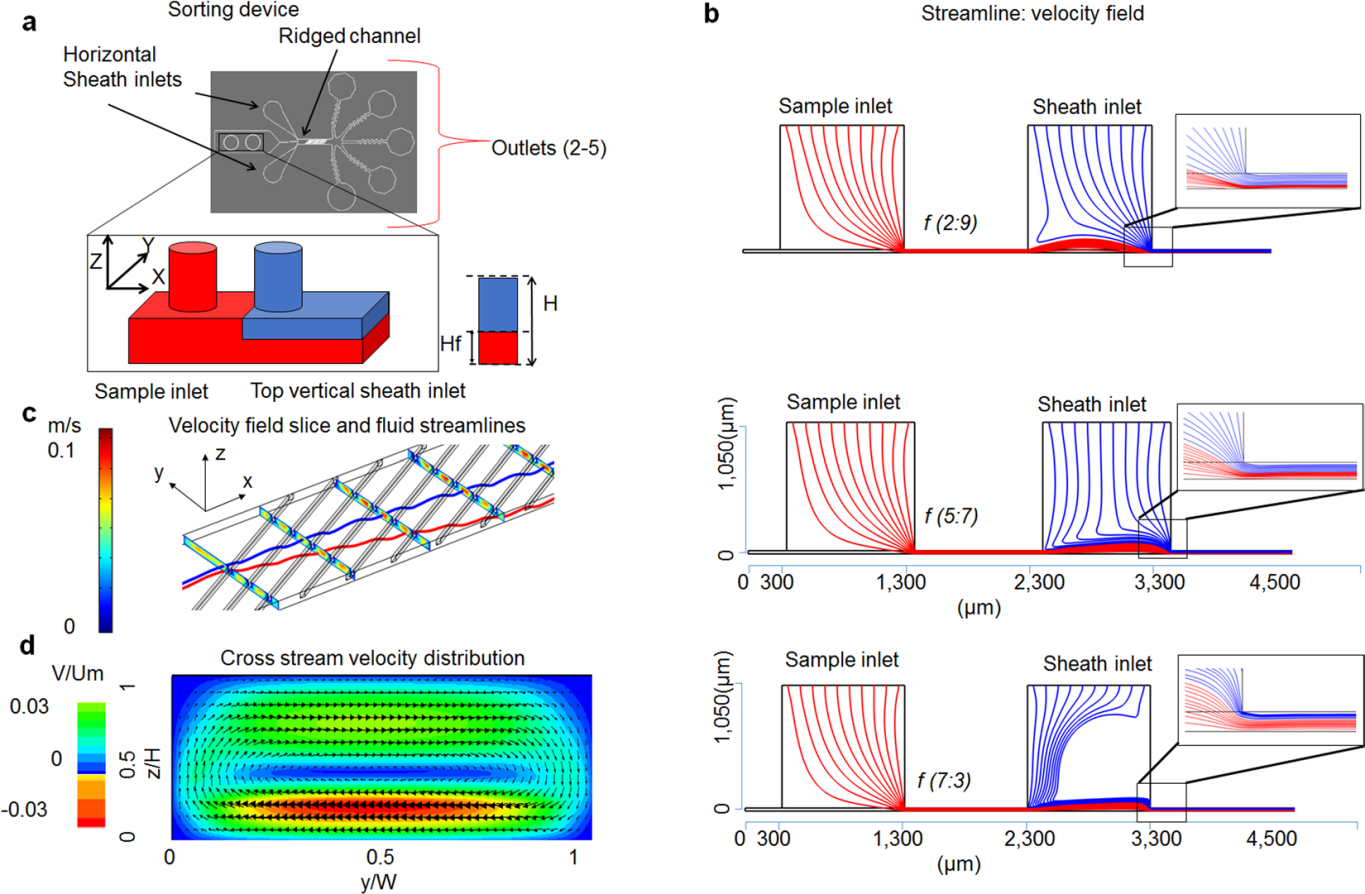
Device Working Principle: (**a**) Image of the sorting device to control the z-position of the particles as they enter the ridged channel using vertical sheath. H and Hf are the heights of the channel and focused streamline respectively. (**b**) COMSOL simulations for different sample to sheath flow rate ratio (*f*). As the vertical sheath flow rate increases the sample flow streamlines are pushed down. (**c**) COMSOL simulations show the velocity field under the ridge and the lateral deviation of streamlines as they enter the ridged part of channel at different heights. (**d**) Cross-stream velocity distribution.

Size based separation and sorting of microparticles and cells improves a variety of biomedical processing steps used in diagnostics and pharmaceutical purification and the study of cell biology^1–5^. Unfortunately, commercially available size based sorting techniques have disadvantages including being labor intensive, deficient in accuracy, and high in cost^6^. A variety of microfluidics approaches have been applied to this problem, including field-flow fractionation^7–12^, and active separation methods which utilize forces from external sources such as dielectric, magnetic, acoustic and optical manipulation^13–21^. Additional passive methods use hydrodynamic effects induced by channel geometry and microstructure^22–25^, membrane filters (MF)^26,27^, deterministic lateral displacement devices (DLD)^28^, and inertial microfluidics^22,29–35^. Inertial microfluidics (IM) features high accuracy and high throughputs comparable to MF^31–33^. An advantage of methods such as IM is use of continuous separation without any external control^22,24,36^. Inertial hydrodynamic methods also utilize non-linear inertial effects in microfluidic channels that provoke cross-stream migration of solid particles. One disadvantage of these methods is that the multiple equilibrium positions of particles in the z-direction^37^ may lead to inaccurate spatial trajectories during separation. Another problem of inertial sorting is that the size-dependent trajectory differences of particles is relatively small and is comparable to particle size. One approach to increase spatial separation in trajectories is using a combination of inertial particle focusing and cross-stream Dean flows generated by channel curvature^29,38,39^. This method, however, requires the use of relatively large spiral microchannels that may be difficult to integrate in lab-on-a-chip devices and employ in parallel settings. Alternatively, continuous size separation devices have used periodically arranged diagonal ridges to create helical flow fields, which leverage transverse flow fields to amplify minute inertial effects and alter the particle trajectory in a size-dependent manner^40–45^. The diagonal ridges create a vortex in which the fluid near the bottom of the channel is transported in the negative y direction, whereas the fluid located near top wall, moves in the positive y direction (lateral direction is along y -axis). The cross-sectional flow pattern is illustrated in Fig. 1d. Thus small vertical displacements of particles caused by size differences can be amplified by larger transverse displacements. However an unsolved problem of size-based sorting by transverse flows is that inaccuracies in particle heights at the entrance lead to variations in particle trajectories.

We demonstrate a novel approach to continuous size-dependent separation of particles that uses a vertical sheath to focus particles to a consistent and optimal position within hydrodynamic flows for enhanced size-dependent enrichment. The separation device consists of a microchannel with periodically arranged diagonal ridges that create helical flow fields. Particles are located to the bottom of the microchannel using vertical focusing, to be then exposed to secondary flows created by diagonal ridges. The incorporation of vertical sheath solves the uncontrolled variation in particle height that leads to variation in transverse flow and thus different trajectory. The height-dependent secondary flows then cause particles with different sizes to migrate transversely with unique trajectories. We use a novel 3D imaging approach^46,47^ to demonstrate that variability of vertical position affects particle trajectories in a manner detrimental to accurate particle sorting. By incorporating z-axis (vertical direction or channel height is along z-axis) focusing of the sample inlet so as to position all particles to the bottom of the channel, we optimize particle exposure to transverse flow fields for size-dependent sorting. With this key innovation we substantially improve the efficiency and accuracy of size based sorting.

Using the new device we have demonstrated the ability to enrich large particles 8-fold and small particles 35-fold. We demonstrate a form of leukapheresis in which leukocytes are separated from whole blood samples—an essential step to reduce the white blood cell count in treating leukemic patients as well as to retrieve blood stem cells in patient with hematologic malignancies^48,49^. We demonstrate size based leukapheresis which offers higher or comparable throughput than other cutting edge techniques and with high recovery and no pre or post processing of samples for analysis and isolation.

## Results and Discussion

**Figure 2:**
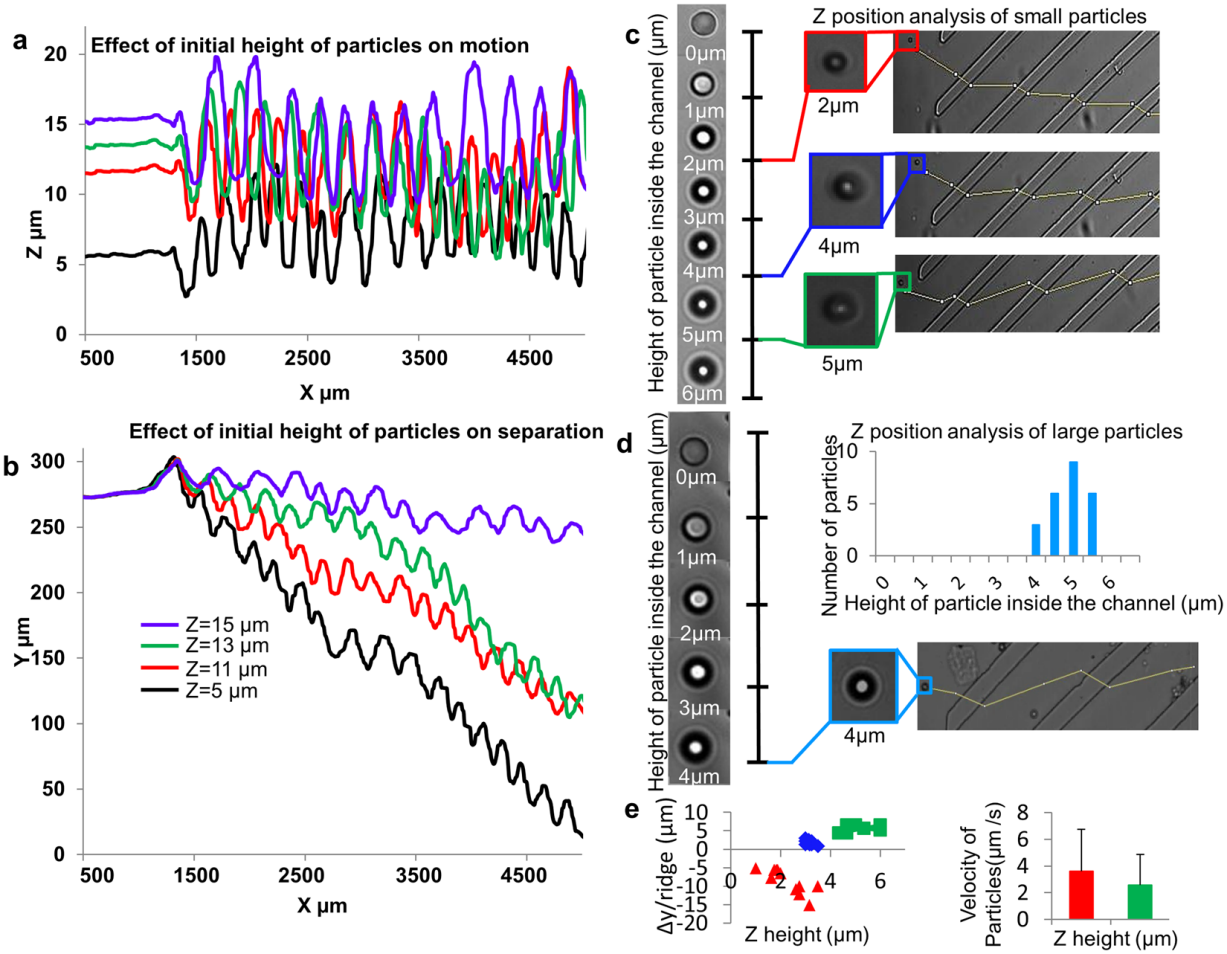
Demonstration of height dependency of lateral positions of particles in a ridged channel: (**a** and **b**) Simulated trajectories showing particles released at different height in a ridged channel will follow different lateral streamlines. (**c**) Experimental trajectories of small particles at different heights. The scale on left shows the reference images of 4 µm particles at different heights inside the channel. The trajectories on the right shows the 4 µm particles at different height which is determined by comparing it to the reference scale on left. (**d**) Experimental trajectories of large particles at different heights. The reference scale for 7 µm particles is shown on left and the trajectory of 7 µm at 4 µm heights on right. The histogram on top right shows that most of 7 µm particles are at a height range of 4 to5.5 µm inside the microchannel. (**e**) Left figure shows Δy/ridge of 4 µm particles at different height in Fig. 2c and right shows their velocity inside the channel.

Figure 1a shows the schematic of the vertical focusing device, consisting of a microchannel with diagonal ridges decorating the top of the channel. Four inlets provide sheath focusing of the sample, two for horizontal and two for the vertical directions. The distance between the ridge and bottom wall of the channel is defined as gap size *Hg*. Particles are considered small if their diameter is less or equal to half the gap size and are considered large if their diameter is greater than half the gap size. By varying the ratio of sample to vertical sheath flow rates, *f*, streamlines could be focused vertically as shown in in the simulation in Fig. 1b. For these simulations we have assumed point particles and that the particles follow the streamlines. The results indicate that as we decrease *f*, the focused sample streamlines are positioned to the bottom of the channel. While optimizing the inlet design we examined the critical parameters including the distance between the sample and vertical sheath inlets and their alignment, as the former can increase the negative pressure from the vertical sheath to sample inlet and convolute the particles within the inlet sample flow. The accurate alignment of the sample and vertical sheath inlets ensures no particle can escape the vertical sheath focusing due to inlet misalignment. Figure 1c shows the simulated streamlines at different heights in the device. The diagonal ridges within the channel create helical flow fields and particles at different vertical heights result in different transverse displacements. Figure 1d shows the cross-stream velocity distribution in a channel with ridged walls (see Methods). The velocity is averaged over one period of the wall structure. The arrows show the direction of fluid velocity, whereas the color represents the normalized magnitude of lateral fluid velocity. Note that the diagonal ridges create circulatory flows that transport fluid to the positive y direction in the channel mid plane and near top wall and to the negative y direction close to the bottom wall. Supplementary Fig. S1 which is generated using COMSOL (see Methods) further shows a single velocity slice and direction of flow under and outside the ridge. In the absence of vertical focusing of the particles, differences in particle positioning exposes each particle to different secondary flows. Additionally, the height-dependent secondary flows also cause particles with different sizes to migrate transversely with unique trajectories^43^. Our simulation results in Fig. 2a and b and experimental data in Fig. 2c,d reveal that microparticles with different sizes separate laterally as they flow in the ridged microchannel, using the separation device with 8 µm gap size to pairwise study the behavior of 7 µm and 4 µm particles^42,46,47^. We demonstrate that there exists a z-position dependent phenomenon which leads to dispersity of smaller particle trajectories. As shown in Fig. 2c, the smaller particles (4 µm in diameter) entering the ridged part of the channel arrive at different vertical positions as determined from out-of-focus images acquired at a constant focal plane. Hence smaller particles at different z-positions, as indicated by different color markers (red for 2 µm, blue for 4 µm, and green for 5 µm heights) move in either positive or negative y-direction trajectories. In contrast, Fig. 2d shows that the larger particles (7 µm diameter) entering the ridged part of the channel uniformly remain in focus at same vertical height and move in the positive y-direction. The histogram of large particle heights in Fig. 2d shows that unlike the smaller 4 µm size particles, most of the larger 7 µm particles enter the ridged channel at same height due to steric hindrance of the ridged features. Figure 2e shows the plot between the Δy/ridge (lateral displacement per ridge) and the diameter of bright diffraction ring, a surrogate for the height of the particle inside the channel using calibrated images of particles at different heights to determine the z position^46,47^. That particles of different heights move with different transverse trajectories results from the vortex flow fields created by the diagonal ridge, such that the fluid at the channel center is transported in the positive y direction, whereas the fluid located near the vertical channel walls moves in the negative y direction as shown by computational fluid dynamics simulations^43^. The particles at different z-positions not only move in different directions but also with different speeds (Fig. 2e), as is expected from the simulated height-dependent velocity gradient^43^. This effect is harnessed to create the separation among particles with different z positions. Supplementary videos S1 and S2 show particles (4 µm) entering the ridged channel at different heights and move in opposite lateral directions.

**Figure 3:**
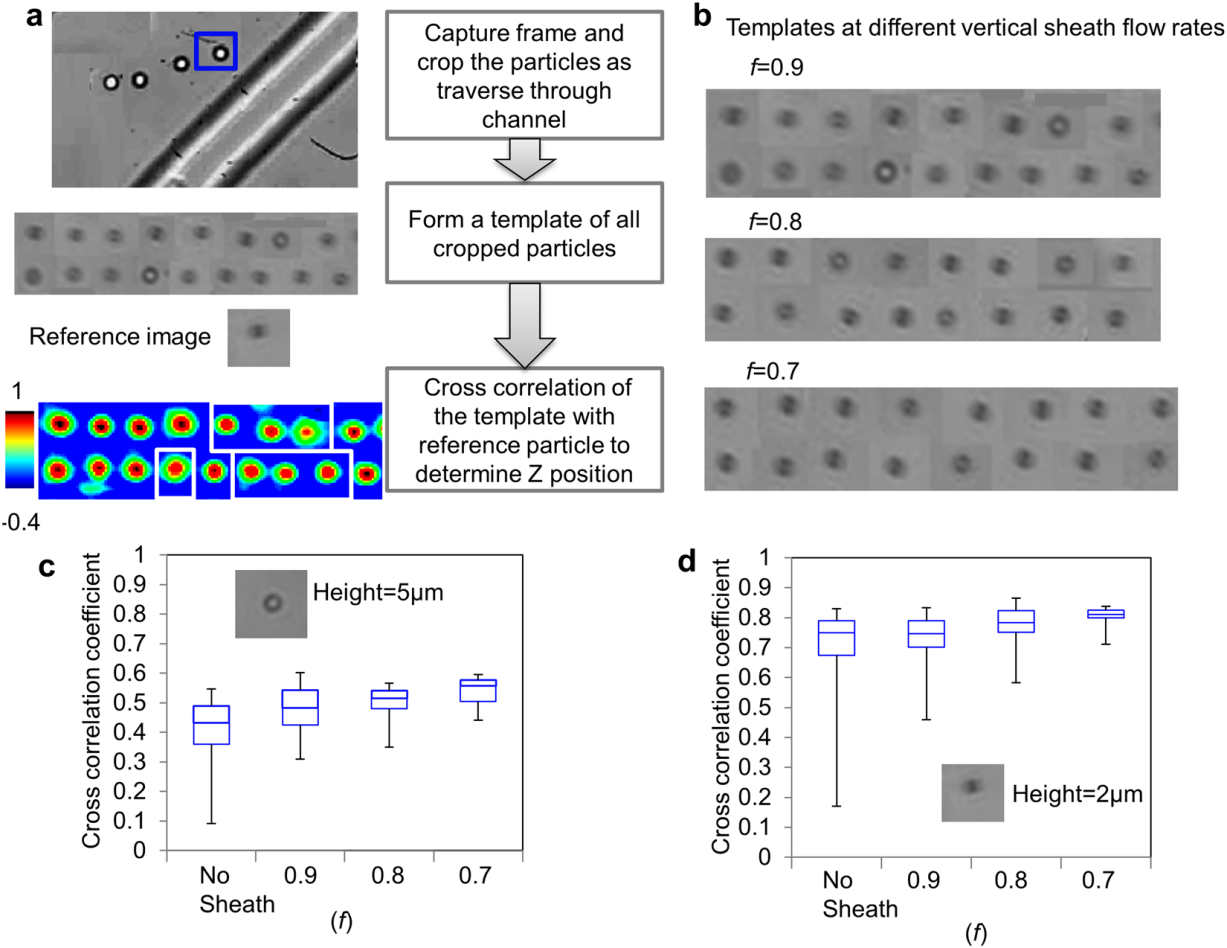
Experimental determination of particle height: (**a**) Algorithm showing how height of particles is estimated using cross correlation with a reference image at a known height. (**b**) Templates showing the particles entering the ridged part of the channel at different vertical sheath flow rates. (**c**) Cross correlation data estimating the height of the particles at different vertical sheath flow rates by comparing the templates in Fig. 3b with reference image at height 5 µm and (**d**) 2 µm.

The goal of the vertical sheath is to position all small particles in the channel to the same z position such that they follow the same trajectory. To mathematically and experimentally determine the height of smaller particles (4 µm) entering the ridged part of the channel after vertical sheath focus, we use theoretical model for predicting the height of two-dimensional hydrodynamically focused streams in rectangular microchannels (details can be found in Supplementary Fig. S2) and video microscopy to examine the vertical position of particles. For video microscopy, still-frame images of particles are cropped from the videos recorded at different sample to vertical sheath rates of *f* = 0.9, 0.8, and 0.7. Figure 3a describes the procedure we used to estimate the z position of particle inside the channel. Each particle in the channel is compared to two reference images for the cross correlation analysis at a vertical focal plane of 5 µm and 2 µm. The templates at different *f* are shown in Fig. 3b. The particles with square boxes in calculated cross correlation template in Fig. 3a show that they are at different z-position as compare to the z position of the reference image. The cross correlation coefficient calculated from the algorithm in Fig. 3a is plotted in Fig. 3c,d. As the flow rate of vertical sheath is increased, the particles are positioned more narrowly to the 2 µm z-position, validating the focusing procedure. This result suggests increase in the enrichment of large particles with increased flow rate of vertical sheath results as more small particles confined to bottom of the channel move in opposite y-direction compared to large particles. Supplementary videos S3 and S4 show the 2 µm particles entering the ridged part of the channel without and with vertical sheath respectively. In case of with vertical sheath, particles are more focused at single z position as all have same diffraction ring pattern.

**Figure 4:**
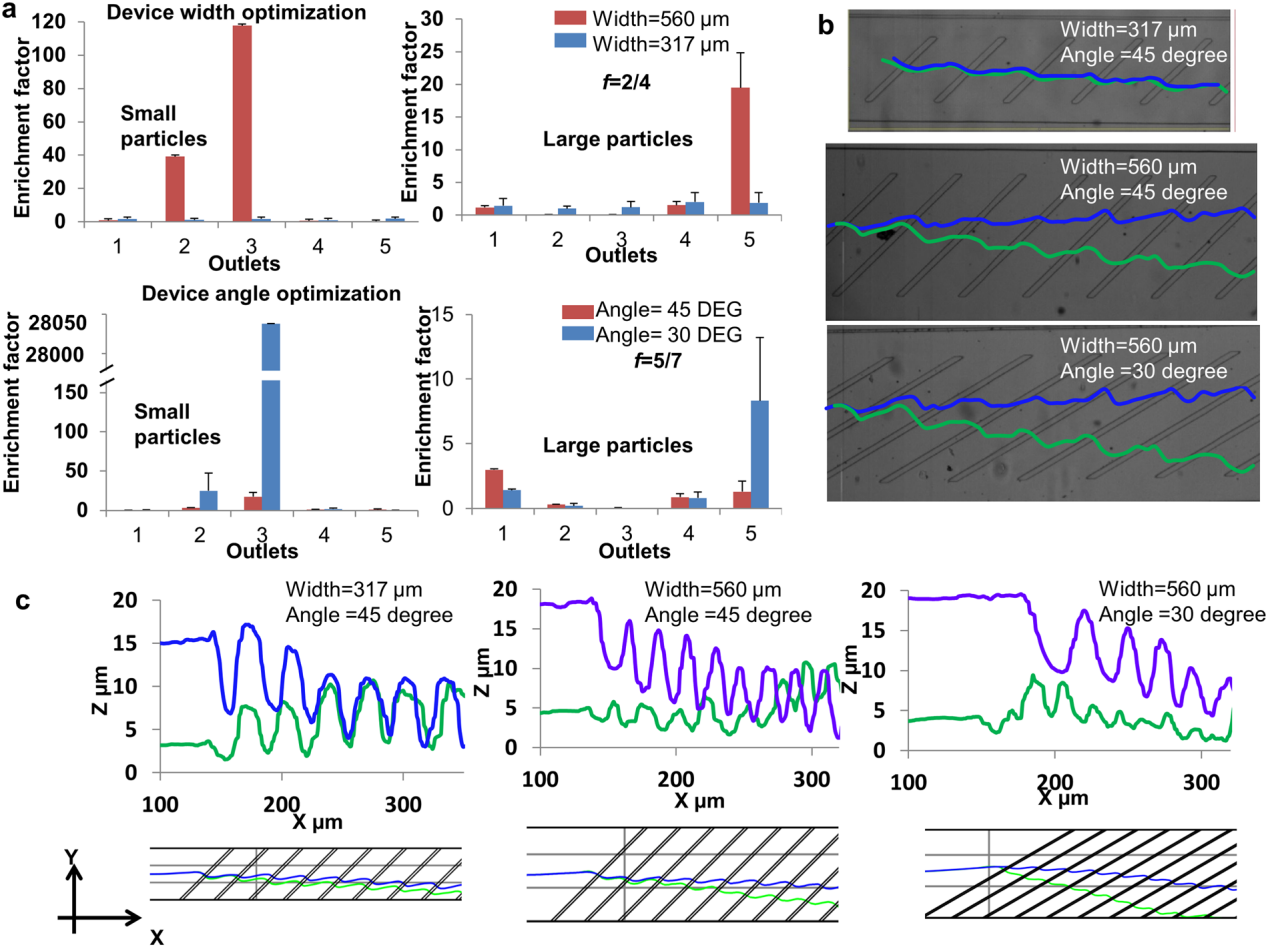
Device geometry optimization: Small and large particle enrichment factors for (**a**) width optimization (top) and angle optimization (bottom). Enrichment factor is the ratio of (targeted particles/non-targeted particles at outlet) to (targeted particles/non-targeted particles at inlet).Error bars represent standard deviation from repeated experiments. Each experiment is repeated 5 times. (**b**) Shows the experimental and (**c**) simulated particle trajectories for different combinations of channel widths and ridge angles.

For this study we have optimized our device design for channel width and inclination angle of ridges for a given channel length for pairwise sorting of 2 µm and 7 µm particles. Enrichment suffers due to a reversal of particle trajectories that occurs once particles reach the channel walls in an unoptimized design, as shown in Supplementary Fig. S3. This effect is due to the circulating velocity vortex formed by diagonal ridges inside the channel, which can be solved by reducing the channel length to avoid the reversal of particle translation due to circulating flow at the edges of the channel for a given width. To study the optimal width, we tested channel widths of 560 and 317 µm with standard 20 µm ridge width and the distance between the two consecutive ridges is 180 µm and ridge inclination angle of 45 degree. For inclination angle study, 45 degree and 30 degree angles of ridges were tested with the 560 µm channel width. Figure 4a shows the enrichment factor (the enrichment factor is defined as the ratio of targeted to non-targeted particles at outlet and inlet) of small and large particles at *f* = 0.5. The smaller width channel device has significantly lower enrichment as compare to 560 µm channel width device. Increased enrichment with channel width can be related to more prominent circulation flows that develop in wider microchannel increasing the relative size based lateral displacement. This can also be seen in experimental data in Fig. 4b and can also be confirmed from simulated trajectories in Fig. 4c. Due to smaller difference in lateral velocity there is not much separation between 2 µm and 7 µm particle trajectories which led to the decrease in relative depth difference between two sized particles as they move along the channel, hence decreasing the efficiency of the device. Reynolds number for a microchannel is given by Re = *u* ρH/µ, where µ is the fluid viscosity, ρ is the density, *H* is the height of channel and *u* is the average velocity. Reynolds numbers for 560 and 317 µm channel widths are 5.3 and 9.6 and channel average velocities are 0.216 m/s and 0.383 m/s, respectively. The enrichment factors for different inclination angles show that 30 degree device has significantly high enrichment and almost 100% purity for small particles. This high purity can be explained by examination of the experimental trajectories. Small and large particles experience opposing velocity fields, as seen in Fig. 2e, at same y position inside the channel in case of 30 degree device and hence are separated quickly with relatively high resolution.

**Figure 5:**
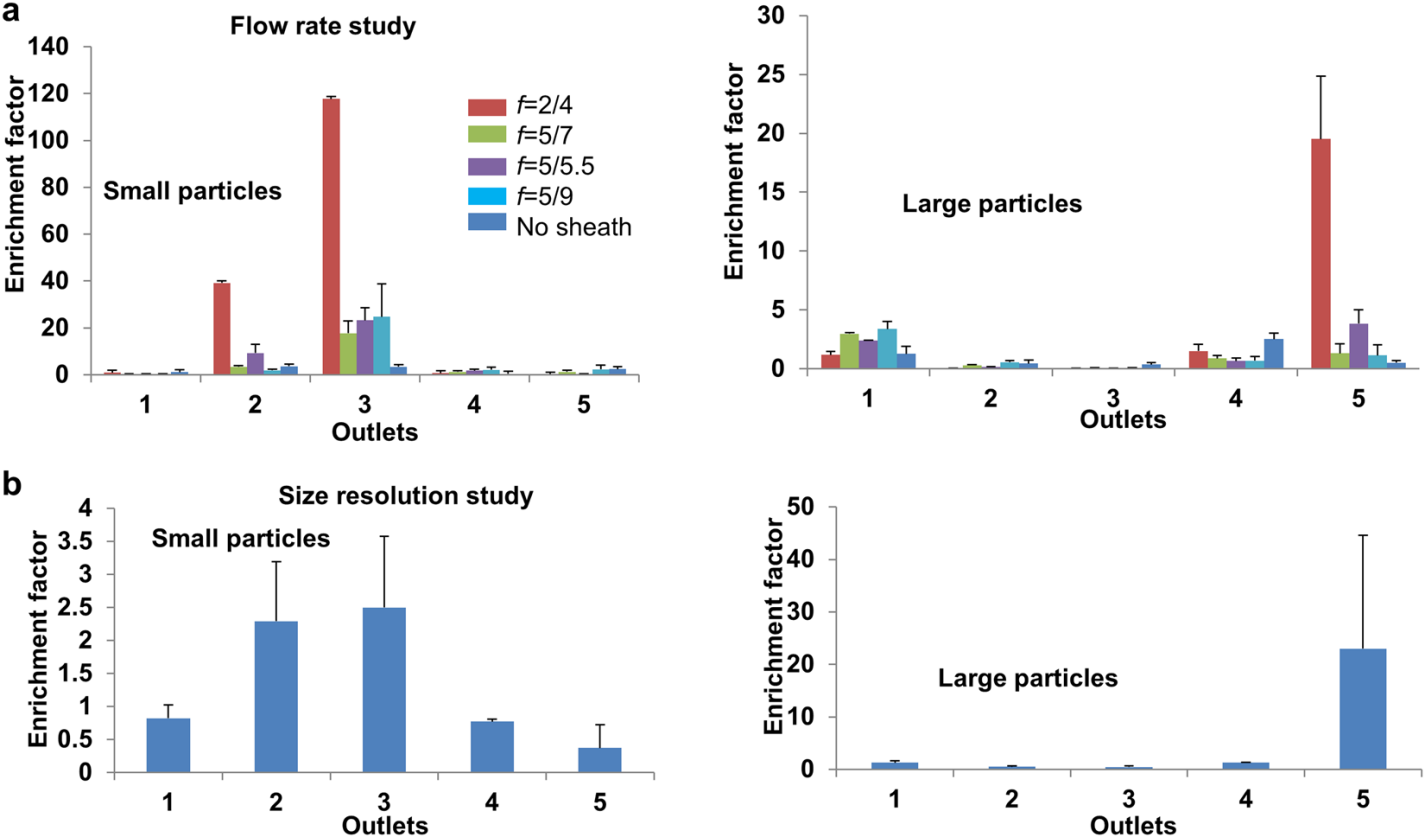
Device flow rate optimization and sorting resolution study: Small and large particle enrichment factors for (**a**) Different flow rates with and without vertical sheath study. The gap size for these studies was 8 µm and the particles were 7 µm and 2 µm. (**b**) Sorting particles with different size differences to evaluate sorting resolution. The gap size is 10.5 µm and the particle sizes are 9.94 µm and 7 µm with *f* equal to 5/9. Error bars represent standard deviation from repeated experiments. Each experiment is repeated 5 times.

We have also optimized the vertical sheath flow rate ratio for best sorting efficiency. The estimated height of small particles was calculated using the model described in Supplementary Fig. S2 for different flow rate ratios. The device design used was 560 µm in width and 45 degree inclination angle. The gap size was 8 µm. Figure 5a shows the enrichment of small (117.8) and large (19.5) particles was highest at *f* = 0.5.

Figure 5a also shows the improvement of sorting enrichment with and without vertical sheath for 8 µm gap size, 560 µm width and 45 degree inclination angle. Small particles are enriched to 117.8 fold from 3.4 with purity of 99.9% and large particles are enriched to 19.5 fold from 2.5 with purity of 70% after incorporating vertical sheath focusing. The purity of large particles in the large particle outlet is less as compared to >99% purity of small particle at small particle outlet that decrease the enrichment of large particles. It is because large particles show decreased transverse displacement per unit length due to their exposure to a velocity field with a small net magnitude compare to vertically focused small particles.

The resolution of sorting was also studied using a 10.5 µm gap size device with 560 µm width and 45 degree ridge inclination angle. Particles of diameter 9.94 µm and 7 µm were sorted with an enrichment of 23 and purity of 90% for large particles and 2.4 and 99% for small particles as shown in Fig. 5b. The throughput of the device with a single channel was 2.8 × 10^6^ particles per minute which is superior as compared to many recent microfluidic studies (Supplementary Table S1).

### Depletion of White Blood Cells (WBCs) Leukapheresis

**Figure 6:**
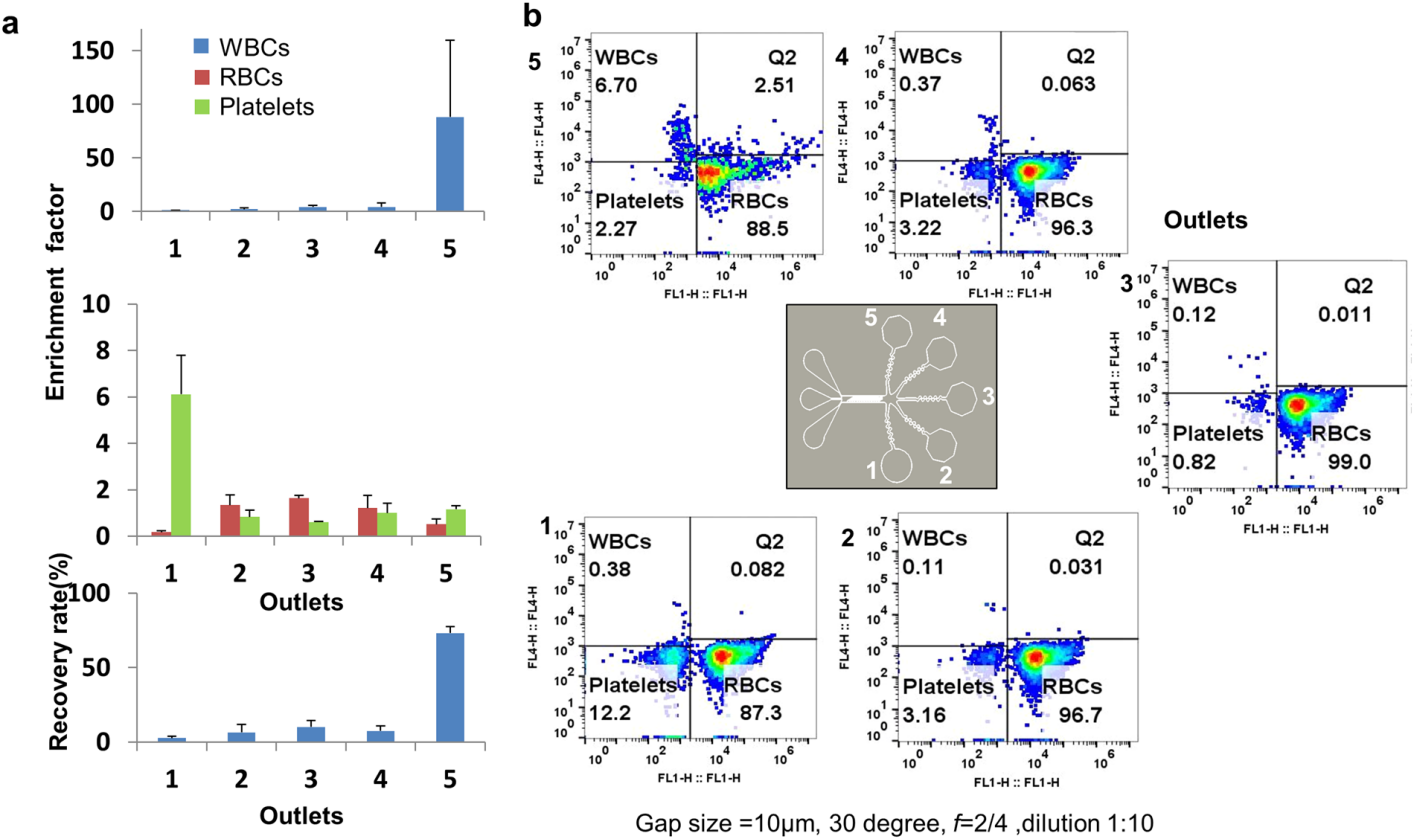
Leukapheresis: (**a**) Enrichment factors for WBCs (top) and platelets and RBCs (middle) and recovery rate of WBCs (bottom). Recovery rate is defined as the % of WBCs at targeted outlet to total number of WBCs collected. (**b**) Flow cytometer characterization of WBC sorting. Error bars represent standard deviation from repeated experiments. Sorting experiments were repeated 5 times.

The design used for this application has a gap size of 10.5 µm with channel width of 560 µm and ridge inclination angle of 30 degrees. A 10.5 µm gap size was selected as red blood cells and platelets are in the range of 2–8 µm and white blood cells are greater than 8 µm. Hence, WBCs are sorted and collected at the top outlet. The throughput is calculated for the blood sample (including RBCs, WBCs and platelets). The WBCs were removed at a depletion of 87 fold and at a high throughput of 0.1 × 10^8^ cells per minute with recovery of 73% as shown in Fig. 6. Cells were analyzed with and without microfluidic processing as shown in Supplementary Fig. 4. No RBC lysing was observed as cells collected after microfluidic processing were intact. Peak wall shear stress for the device was calculated (see Methods) and was 230 Pa. A maximum threshold shear stress value of RBCs is reported to be 450 Pa^50,51^, thereby confirming that the shear stress is not expected to cause hemolysis.

These results suggest that a microfluidic approach can be developed for leukapheresis with high depletion and recovery rate of WBCs with ease of batch fabrication and low cost, especially if parallelization scaled the throughput and any toxicity of PDMS is addressed. Removal of leukocytes before performing a blood transfusion minimizes transmission of cell-associated infectious agents (cytomegalovirus, herpes virus, human T-cell lymphoma virus), graft rejection rates and prevents febrile transfusion reactions^52^. Conventional macroscale WBCs sorting techniques include centrifugation, apheresis, chemical lysis, and antibody based techniques including fluorescence-activated cell sorting (FACS) and magnetic-activated cell sorting (MACS)^53–60^ all face drawbacks. Centrifugation is commercially available, easy to conduct and viability of fractionated blood cells is very high but it does not allow for high separation resolution, is an open process exposed to contamination, and requires milliliters of blood for analysis and dedicated labs and trained users are pre -requisites. Microscale flow-based protocols such as FACS or solid-state immobilization and capture of cells using MACS offers advantages like high specificity, high-throughput, commercial availability but are expensive and cannot reliably handle small numbers of cells. Our device operates simply with a small foot print, label-free, and offers a clog-free and self-cleaning platform^61^ with high throughput and high purity and enrichment. Comparison with other cutting edge technologies can be found in Supplementary Tables S1 and S2. The proposed z-focusing approach may be extended to separate nanoparticles by using smaller gap size and using an optimized vertical flow rate ratio. Due to its small dimensions, the proposed device can easily be implemented with parallel channels^32^ to increase throughput.

## Conclusions

We have described a novel microfluidic device capable of improved purity of size-dependent separation of particles by positioning particles at the optimal position in three-dimensional space to exaggerate the size-dependent trajectory differences. The unique aspect of this sorting design is the use of three-dimensional focusing of the sample to a position that optimizes the differential secondary flows. Using vertical focusing, we are able to separate 2 µm particles from 7 µm with a substantial improvement in enrichment of sorted number density in comparison to unfocused flow. We demonstrate 8-fold increase of the purity of the larger particles (from 2.5 to 19.5–fold enrichment factor) and a 35–fold increase in the purity of smaller particles (from 3.4–fold to 117.8–fold). This is the first time that a device with 3D focusing has been reported to improve the purity of samples using size sorting. The proposed technology can further be improved for higher purity enrichment of particles by modifying channel geometry and gap size^43^. It can also be integrated with separation techniques using different biomarkers like stiffness and viscoelastic deformation^44,61^.

## Methods

### Experimental section

#### Microfluidic Device Fabrication

The microfluidic devices with different gap size were fabricated by replica molding Polydimethylsiloxane (PDMS) (Sylgard 184 Dow Corning Corp) on a permanent mold. The mold is made from SU-8 2007 using a two mask photolithography process. The mold dimensions were characterized with profilometry (Dektak 150 profiler) and verified with confocal microscopy imaging (Olympus LEXT). Uncured PDMS was mixed in a 10:1 ratio of elastomer to curing agent, then poured onto the SU-8 molds to a thickness of 1 cm and cured in an oven at 60 °C for 6 hours. The cured PDMS layer was cut and peeled off the mold and inlet and outlet holes were formed with a 1 mm biopsy punch. The PDMS device was treated with oxygen plasma (Harrick plasma cleaner PDS 32G) for 2 minutes then bonded to a glass microscope slide. The separation device consists of a microchannel with periodically arranged diagonal ridges. We designed devices with two sets of dimensions: 5.5 mm channel length and 560 µm channel width and 25 ridges and 2.5 mm channel length and 317 µm channel width and 11 ridges. All devices have 22 µm channel height. The ridges are 20 µm wide and distance between two consecutive ridges is 180 µm. The ridges are at the top of the microchannel and are inclined at either a 30 or 45 degrees.

### Sample Preparation

Spherical polystyrene particles of 2.19, 4, 7.06 and 9.94 µm diameters (Bangs Laboratories Inc.) were used in the flow experiments. Dispersions were prepared in phosphate buffered saline (PBS) solution with a concentration of approximately 2 × 10^4^ particles/µL. To prevent bead adhesion, 0.01% v/v Tween 20 was added to flow media. For white blood cell separation, discarded fresh human blood was withdrawn from healthy donors using protocols approved by the Georgia Institute of Technology Institute Review Board^43^. Blood samples were collected in citrate coated vials and used immediately after collection. The blood sample was diluted 1:10 in PBS before separation experiments.

### Experimental Setup

Syringe pumps (PHD 2000 Harvard Apparatus) were used to control the flow rates of the sample and sheath flows for the experiment. Dispersed particles were contained in a plastic syringe and infused into the microfluidic device through polyethylene tubes. The particle flow within the microfluidic device was observed with an inverted microscope (Nikon Eclipse Ti) and the videos were recorded using a high-speed camera (Phantom v7.3 Vision Research). Both x-y positions of particles were collected as well as the vertical position of the particles using an out-of-focus tracking algorithm developed previously^47^. This algorithm matches each particle image to a reference library of identical particles imaged at various degrees of defocus in order to determine the vertical position of the particle. In order to accurately capture the cell trajectories with sufficient accuracy to determine the vertical position, we operated the high speed camera at a minimum of 3000 frames per second with a minimum resolution of 640 by 480 pixels for all videos and images. Separated particles and cells were collected at the outlets and were analyzed with an Accuri C6 flow cytometer (BD Biosciences). Purity of leukocytes is determined using the antibody cocktail - anti CD45 -APC anti CD61-PE, and anti CD235a-FITC (GlycophorinA) from Biolegend, following manufacturer’s protocols.

### Finite Element Simulation

Finite element simulations were performed using COMSOL Multiphysics software (COMSOL Inc., Burlington, MA). Simulations were performed for channel widths of 317 and 560 µm and ridge inclination angles of 30 and 45 degrees. PDMS was selected as material of interfacing structure. Sample to vertical sheath flow rates ratios of 0.22, 0.71 and 2.3 were used in the simulation to determine their effect on sample vertical confinement. The flow profiles in the channel were obtained by solving the Navier-Stokes equations for incompressible fluid using Fluid-Structure Interaction physics. At the outlet, the pressure was set to zero with no viscous stress on the boundary. Due to low Reynolds’s number of fluid, it was assumed that the suspended particles would follow the fluid streamlines.

Three-dimensional computational fluid dynamic simulations were performed to obtain cross-stream velocity distribution in the channel. A pressure-driven flow in a periodic microfluidic channel of height *H* and width *W* was considered.The top channel walls were lined with symmetrical solid ridges. In current implementation, the channel was periodic in the *x* direction. A constant pressure gradient is imposed in the negative *x* direction via a uniform body force to create a Poiseuille flow in the channel. The velocity is averaged over one period of the wall structure.

### Wall Shear Stress Calculation

The maximum wall shear stress inside the channel is estimated (assuming Poiseuille flow) using relation τ = 6 μQ/*WHg* ^2^, where τ is the wall shear stress, *W* and *Hg* are channel width and gap size respectively and µ is viscosity of the buffer^62^.

## Electronic supplementary material

Supplementary Information

Supplementary Video 1

Supplementary Video 2

Supplementary Video 3

Supplementary Video 4

## References

[1] Kallioniemi O-P, Wagner U, Kononen J, Sauter G (2001). Tissue microarray technology for high-throughput molecular profiling of cancer. Human Molecular Genetics.

[2] Müller V (2012). Prognostic impact of circulating tumor cells assessed with the CellSearch System™ and AdnaTest Breast™ in metastatic breast cancer patients: the DETECT study. Breast Cancer Research: BCR.

[3] Nomura, S., Ozaki, Y. & Ikeda, Y. Function and role of microparticles in various clinical settings. *Thrombosis Research* **123**, 8–23, 10.1016/j.thromres.2008.06.006. 10.1016/j.thromres.2008.06.006 18667228

[4] Parkinson DR (2012). Considerations in the development of circulating tumor cell technology for clinical use. Journal of Translational Medicine.

[5] Sethu P, Sin A, Toner M (2006). Microfluidic diffusive filter for apheresis (leukapheresis). Lab on a Chip.

[6] Davey HM, Kell DB (1996). Flow cytometry and cell sorting of heterogeneous microbial populations: the importance of single-cell analyses. Microbiological reviews.

[7] Battu S, Roux A, Delebasee S, Bosgiraud C, Cardot PJ (2001). Sedimentation field-flow fractionation device cleaning, decontamination and sterilization procedures for cellular analysis. Journal of Chromatography B: Biomedical Sciences and Applications.

[8] Bernard A, Paulet B, Colin V, Cardot PJ (1995). Red blood cell separations by gravitational field-flow fractionation: instrumentation and applications. TrAC Trends in Analytical Chemistry.

[9] Metreau JM (1997). Sedimentation field-flow fractionation of cellular species. Analytical Biochemistry.

[10] Reschiglian P (2002). Bacteria sorting by field-flow fractionation. Application to whole-cell Escherichia coli vaccine strains. Analytical Chemistry.

[11] Wang X-B (2000). Cell separation by dielectrophoretic field-flow-fractionation. Analytical Chemistry.

[12] Yang J, Huang Y, Wang X-B, Becker FF, Gascoyne PR (2000). Differential analysis of human leukocytes by dielectrophoretic field-flow-fractionation. Biophysical Journal.

[13] Doh I, Cho Y-H (2005). A continuous cell separation chip using hydrodynamic dielectrophoresis (DEP) process. Sensors and Actuators A: Physical.

[14] Guo F (2015). Reusable acoustic tweezers for disposable devices. Lab on a Chip.

[15] Han K-H, Frazier AB (2006). Paramagnetic capture mode magnetophoretic microseparator for high efficiency blood cell separations. Lab on a Chip.

[16] Kim U, Qian J, Kenrick SA, Daugherty PS, Soh HT (2008). Multitarget dielectrophoresis activated cell sorter. Analytical chemistry.

[17] Pamme N, Wilhelm C (2006). Continuous sorting of magnetic cells via on-chip free-flow magnetophoresis. Lab on a Chip.

[18] Perroud TD (2008). Microfluidic-based cell sorting of Francisella tularensis infected macrophages using optical forces. Analytical Chemistry.

[19] Sarioglu, A. F. *et al*. A microfluidic device for label-free, physical capture of circulating tumor cell clusters. *Nature Methods* (2015). 10.1038/nmeth.3404 PMC4490017 25984697

[20] Wang MM (2005). Microfluidic sorting of mammalian cells by optical force switching. Nature Biotechnology.

[21] Xia N (2006). Combined microfluidic-micromagnetic separation of living cells in continuous flow. Biomedical Microdevices.

[22] Di Carlo D (2009). Inertial microfluidics. Lab on a Chip.

[23] Lee K, Shao H, Weissleder R, Lee H (2015). Acoustic Purification of Extracellular Microvesicles. ACS nano.

[24] Lenshof A, Laurell T (2010). Continuous separation of cells and particles in microfluidic systems. Chemical Society Reviews.

[25] Pamme N (2007). Continuous flow separations in microfluidic devices. Lab Chip.

[26] Wei H (2011). Particle sorting using a porous membrane in a microfluidic device. Lab Chip.

[27] Zheng S (2007). Membrane microfilter device for selective capture, electrolysis and genomic analysis of human circulating tumor cells. Journal Of Chromatography. A.

[28] Huang LR, Cox EC, Austin RH, Sturm JC (2004). Continuous particle separation through deterministic lateral displacement. Science.

[29] Bhagat AAS, Kuntaegowdanahalli SS, Papautsky I (2009). Inertial microfluidics for continuous particle filtration and extraction. Microfluid. Nanofluid..

[30] Chmela E, Tijssen R, Blom MT, Gardeniers HJGE, van den Berg A (2002). A Chip System for Size Separation of Macromolecules and Particles by Hydrodynamic Chromatography. Anal. Chem..

[31] Lee, M. G., Bae, C. Y., Choi, S., Cho, H. & Park, J. High-Throughput Inertial Separation Of Cancer Cells From Human Whole Blood In A Contraction-Expansion Array Microchannel. *Proceedings Of Micro Tas*, 2065–2067 (2011).

[32] Mach AJ, Di Carlo D (2010). Continuous scalable blood filtration device using inertial microfluidics. Biotechnol. Bioeng..

[33] Mach AJ, Kim JH, Arshi A, Hur SC, Di Carlo D (2011). Automated cellular sample preparation using a Centrifuge-on-a-Chip. Lab Chip.

[34] Pamme N (2007). Continuous flow separations in microfluidic devices. Lab on a Chip.

[35] Yamada M, Seki M (2005). Hydrodynamic filtration for on-chip particle concentration and classification utilizing microfluidics. Lab Chip.

[36] Di Carlo D, Edd J, Humphry K, Stone H, Toner M (2009). Particle Segregation and Dynamics in Confined Flows. Phys. Rev. Lett..

[37] Di Carlo D, Irimia D, Tompkins RG, Toner M (2007). Continuous inertial focusing, ordering, and separation of particles in microchannels. Proceedings of the National Academy of Sciences.

[38] Russom A (2009). Differential inertial focusing of particles in curved low–aspect-ratio microchannels. New J. Phys..

[39] Squires TM, Quake SR (2005). Microfluidics: Fluid physics at the nanoliter scale. Rev. Mod. Phys..

[40] Choi S, Ku T, Song S, Choi C, Park J-K (2011). Hydrophoretic high-throughput selection of platelets in physiological shear-stress range. Lab on a Chip.

[41] Choi S, Park J-K (2007). Continuous hydrophoretic separation and sizing of microparticles using slanted obstacles in a microchannel. Lab Chip.

[42] Choi S, Park J-K (2009). Tuneable hydrophoretic separation using elastic deformation of poly(dimethylsiloxane). Lab on a Chip.

[43] Mao W, Alexeev A (2011). Hydrodynamic sorting of microparticles by size in ridged microchannels. Physics of Fluids.

[44] Wang G (2015). Microfluidic cellular enrichment and separation through differences in viscoelastic deformation. Lab on a Chip.

[45] Wang, G., Mao, W., Henegar, C., Alexeev, A. & Sulchek, T. in*ASME* 2012 *Summer Bioengineering Conference*. 241–242 (American Society of Mechanical Engineers).

[46] Peterson SD, Chuang H-S, Wereley ST (2008). Three-dimensional particle tracking using micro-particle image velocimetry hardware. Measurement Science and Technology.

[47] Tasadduq B (2015). Three-dimensional particle tracking in microfluidic channel flow using in and out of focus diffraction. Flow Measurement and Instrumentation.

[48] Buckner D, GRAW RG, Eisel RJ, Henderson ES, Perry S (1969). Leukapheresis* by continuous flow centrifugation (CFC) in patients with chronic myelocytic leukemia (CML). Blood.

[49] Malachowski M, Comenzo R, Hillyer C, Tiegerman K, Berkman E (1992). Large‐volume leukapheresis for peripheral blood stem cell collection in patients with hematologic malignancies. Transfusion.

[50] Paul R (2003). Shear stress related blood damage in laminar couette flow. Artificial organs.

[51] Chang, W., Tzebotich, D., Lee, L. P. & Liepmann, D. In *Microtechnologies in Medicine and Biology*, *1st Annual International*, *Conference On*. 2000. 311–315 (IEEE).

[52] Chu R (1999). Leukocytes in blood transfusion: adverse effects and their prevention. Hong Kong Med J.

[53] Hardwick J (2008). Blood processing. ISBT Science Series.

[54] Kreuger A, Åkerblom O, Högman CF (1975). A Clinical Evaluation of Citrate‐Phosphate‐Dextrose‐Adenine Blood. Vox sanguinis.

[55] Lozada JL, Caplanis N, Proussaefs P, Willardsen J, Kammeyer G (2001). Platelet-rich plasma application in sinus graft surgery: Part I-Background and processing techniques. Journal of Oral Implantology.

[56] Pasqualetti D (2004). Blood component fractionation: manual versus automatic procedures. Transfusion and apheresis science.

[57] Rossi, E. & Simon, T. Rossi’s principles of transfusion medicine. (2009).

[58] Chernyshev AV (2008). Erythrocyte lysis in isotonic solution of ammonium chloride: Theoretical modeling and experimental verification. Journal of theoretical biology.

[59] Bonner W, Hulett H, Sweet R, Herzenberg L (1972). Fluorescence activated cell sorting. Review of Scientific Instruments.

[60] Miltenyi S, Müller W, Weichel W, Radbruch A (1990). High gradient magnetic cell separation with MACS. Cytometry.

[61] Wang G (2013). Stiffness Dependent Separation of Cells in a Microfluidic Device. PLoS ONE.

[62] Bose, S. *et al*. Affinity flow fractionation of cells via transient interactions with asymmetric molecular patterns. *Scientific reports* **3** (2013). 10.1038/srep02329 PMC3728592 23900203

