# Supplementary material for: Enhancing size based size separation through vertical focus microfluidics using secondary flow in a ridged microchannel
Source: Sci Rep. 2017 Dec 12;7:17375. doi: 10.1038/s41598-017-17388-w (PMC5727182; doi:10.1038/s41598-017-17388-w)
Supplement: Supplementary file 1 — Supplementary Information [file 41598_2017_17388_MOESM1_ESM.pdf]

Enhancing size based size separation through vertical focus microfluidics using secondary flow in a ridged microchannel

Bushra Tasadduq,[±, °] Wilbur Lam,[†] Alexander Alexeev,[§] A. Fatih Sarioglu[±, §] and Todd Sulchek[†, §,*]

[±] School of Electrical and Computer Engineering, Georgia Institute of Technology, Atlanta GA

°NED University of Engineering & Technology, Karachi Pakistan

† Wallace H Coulter Department of Biomedical Engineering, Georgia Institute of Technology, Atlanta GA

§ Woodruff School of Mechanical Engineering, Georgia Institute of Technology, Atlanta GA

*Correspondence to todd.sulchek@me.gatech.edu

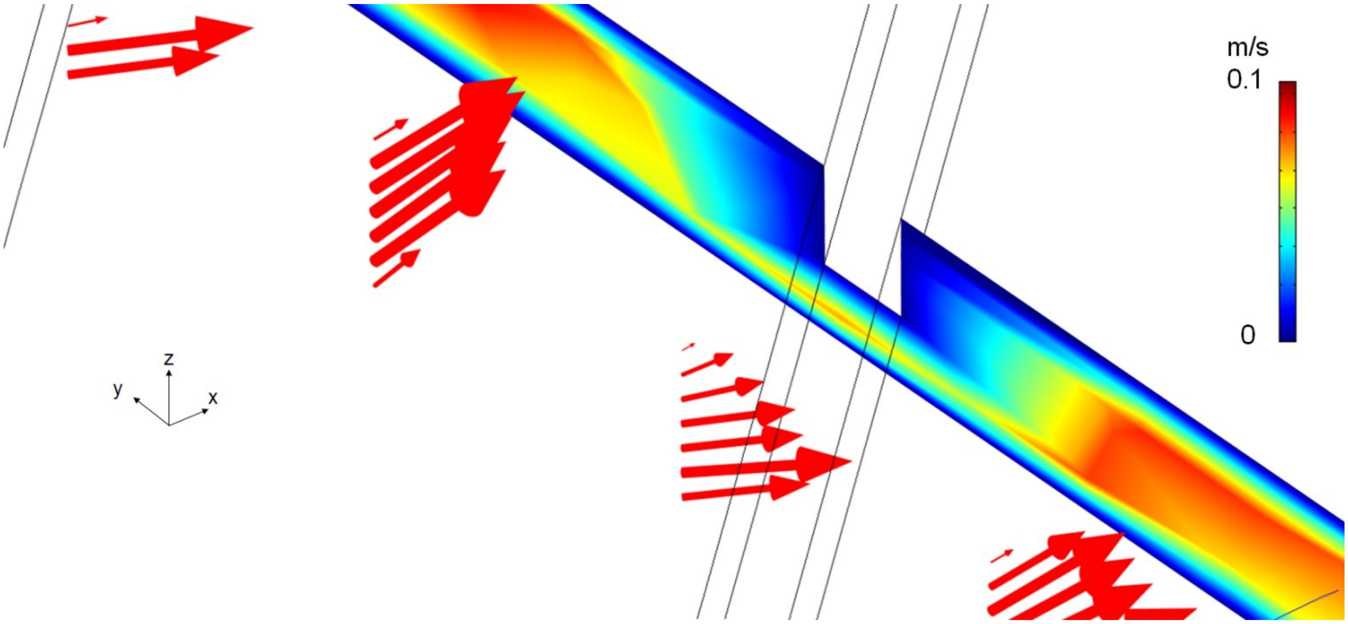

Supplementary Figure S1 Velocity of flow inside the ridged microchannel. Arrows indicate the direction of flow field.

A mathematical model for predicting the height of hydrodynamically focused streams in a rectangular microchannel

The three dimensional hydrodynamic focusing was implemented in a similar manner previously described[1]. The model for predicting the height of 2 D hydrodynamically focused streams in a rectangular microchannel is given in Supplementary Fig. S2**a** and derived as described previously[2]. Following assumptions are made: (i) Flow in the microchannels is steady and laminar. (ii) Fluids are Newtonian. (iii) Fluid has the same density in the inlet, sheath and outlet channel.

a

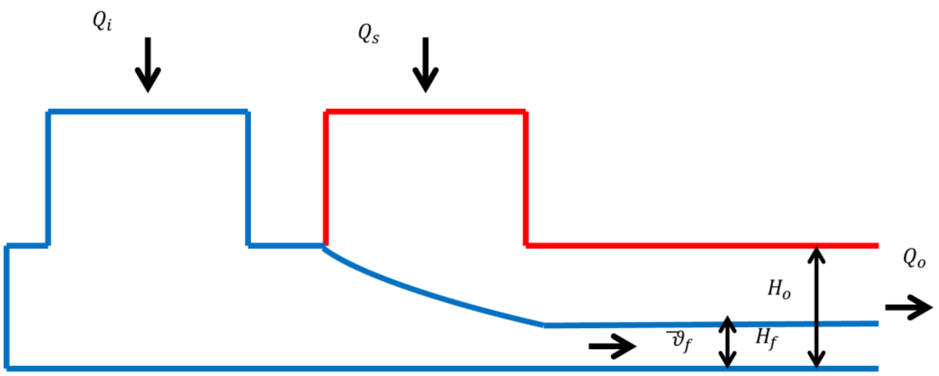

b

$\frac{Q_i}{Q_s}$	H_f(Calculated)μm	H_f(Simulated) μm
7/3(A)	2	2.5
5/6(B)	9	8
1/9(C)	13.9	14.1

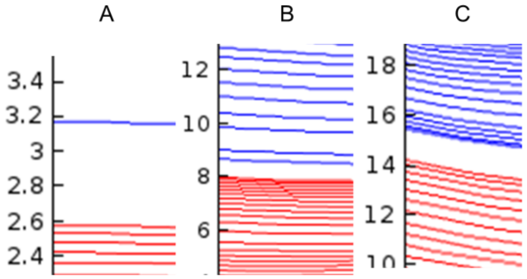

Supplementary Figure S2 Mathematical model for determining the height of focused streamline: **a**) Schematic of the model for determining the height of the focused streamline in a rectangular microchannel. **b**) Calculated and simulated (COMSOL) Hf with cropped images of simulated streamlines on right for multiple cases of *f*.

According to the principle of mass conservation, the amount of fluid passing through the inlet channel must equal to the amount of fluid passing through the dimension of the focused stream, i.e.

$$H_f = \frac{Q_i}{\overline{\vartheta}_f X\, W} \tag{1}$$

H is the height and W is the width of the channel, H_f is hydrodynamically focused stream and Q_i and Q_s are volumetric flow rates of the sample and sheath channel inlets. The total amount of fluid passing through the outlet channel must equal the total amount of fluid supplied from the inlet and sheath,

$$\overline{\vartheta}_o = \frac{Q_i + Q_s}{H X W} \quad (2)$$

Therefore, the relationship between the height of the hydrodynamically focused stream and the volumetric flow rates of the inlet channel and the sheath can be expressed as

$$\frac{H_f}{H} = \frac{Q_i}{(Q_s + Q_i)\gamma} \quad (3)$$

where the velocity ratio $\gamma = \overline{\vartheta}_f / \overline{\vartheta}_o$ is to be found. $\overline{\vartheta}_f$ and $\overline{\vartheta}_o$ are the average flow velocities in the focused stream and the outlet channel, respectively. The Reynolds number is generally very small in microfluidic devices. Since viscous effects dominate in low Reynolds number flows, the entrance length in the microchannel is very short. Therefore, the flow in the outlet channel can be assumed to be fully developed. Hence, the basic equation of the flow is given by

$$\frac{\partial^2 u}{\partial y^2} + \frac{\partial^2 u}{\partial z^2} = \frac{1}{\mu}\frac{dp}{dx} = const \quad (4)$$

where $u(y, z)$, dp/dx and μ are the streamwise velocity, pressure gradient and fluid viscosity in the outlet channel respectively. Imposing the no-slip condition on the channel wall, equation (4) can be solved as

$$u(y,z) = \frac{4W^2}{\mu\pi^3}\left(\frac{-dp}{dx}\right)\sum_{n=0}^{\infty}(-1)^n X \left\{1 - \frac{\cosh[\frac{(2n+1)\pi y}{W}]}{\cosh[\frac{(2n+1)\pi H}{2W}]}\right\}\frac{\cos[\frac{(2n+1)\pi z}{W}]}{(2n+1)^3} \quad (5)$$

Equation (5) is the well-known Poiseuille velocity profile for flow through a rectangular channel. Integrating equation (5) along the y-direction, the streamwise average velocity $\overline{u(z)}$ in the rectangular microchannel can be expressed as

$$\overline{u(z)} = \frac{1}{W}\int_{-\frac{W}{2}}^{\frac{W}{2}} u(y,z)dz = \frac{8W^2}{\mu\pi^4}\left(\frac{-dp}{dx}\right)\sum_{n=0}^{\infty}\frac{1}{(2n+1)^4} X \left\{1 - \frac{\cosh\left[\frac{(2n+1)\pi y}{W}\right]}{\cosh\left[\frac{(2n+1)\pi H}{2W}\right]}\right\} \quad (6)$$

The average velocities in the outlet channel, $\overline{\vartheta}_o$, and the focused stream, $\overline{\vartheta}_f$, can be obtained from equation (6). The velocity ratio, γ, is then given by

$$\gamma = \overline{\vartheta}_f / \overline{\vartheta}_o = \frac{\frac{2}{H_f}\int_0^{H_f/2}\overline{u}(y)dy}{\frac{2}{H_f}\int_0^{H/2}\overline{u}(y)dy} = \frac{\left\{1 - (192W/\pi^5 H_f \sum_{n=0}^{\infty}\frac{1}{(2n+1)^5}\frac{\sinh(2n+1)\pi H_f/2W}{\cosh(2n+1)\pi H_f/2W}\right\}}{\left\{1 - (192W/\pi^5 H \sum_{n=0}^{\infty}\frac{\tanh(2n+1)\pi H/2W}{(2n+1)^5}\right\}} \quad (7)$$

Equation (7) reveals that γ is dependent on the aspect ratio. When aspect ratio $\varepsilon = H/W \rightarrow 0$, a parabolic velocity profile is formed across the channel height and is independent of the position across the channel width (i.e. the velocity profile is 'plug-like' across the channel width). Under such conditions, $\gamma = 1.0$[3] . Table in Supplementary Fig.S2**b** shows the comparison between the theoretical and numerical (COMSOL) models for the height of hydrodynamically focused stream.

From these simulations we found a range of flow rate ratios that positioned particles in a consistent z position range for improved consistency of transverse flow fields and thus improved size sorting.

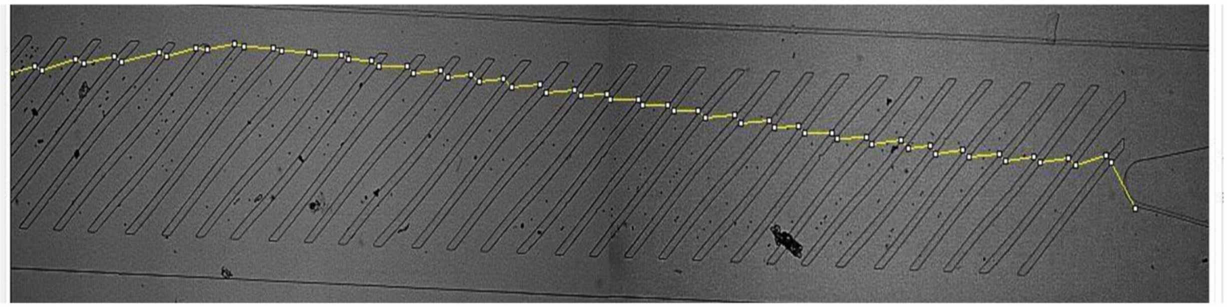

Supplementary Figure S3 Oscillation of small particles in wide channels.

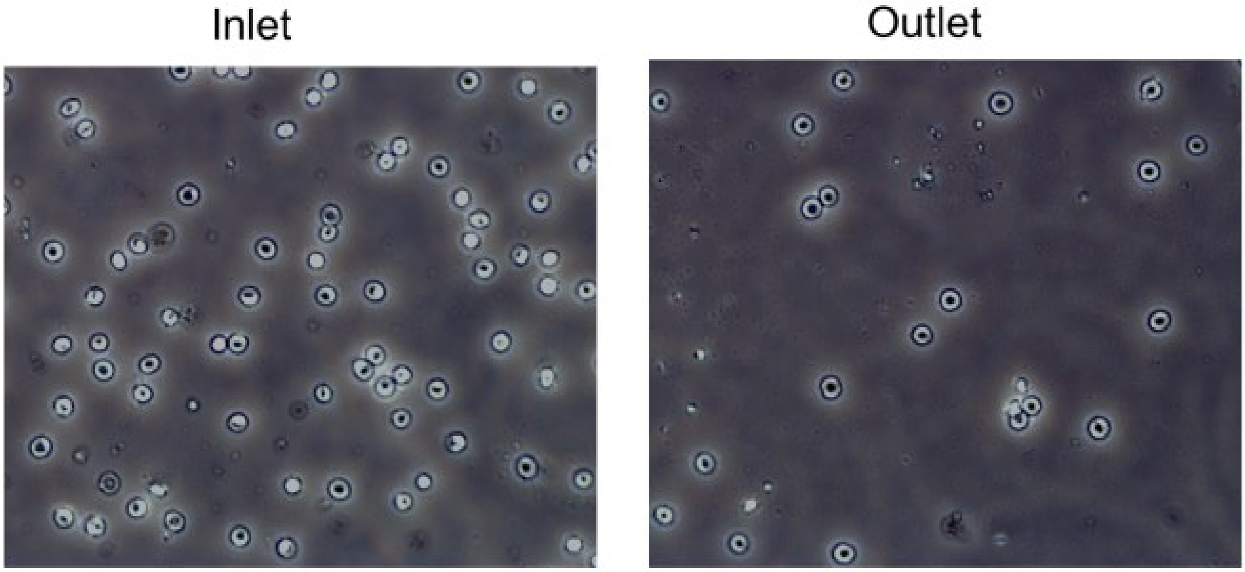

Supplementary Figure S4 No RBCs lysing as cells collected at outlet are intact. For negative control image of cells at inlet is also shown.

Supplementary Table S1: Particle sorting comparison

Reference	*Mechanism*	*Throughput 100*particles/min*	*Enrichment (Large)*	*Enrichment (Small)*	*Resolution =Difference between particle size (μm)*
[4]	Inertial separation	1090	6.38	>10000	5
[5]	Hydrodynamic filtration with no inertia	548	39	>10000	6.9
[5]	Hydrodynamic filtration with no inertia	548	15.6	>10000	5.1
[6]	Inertia and Secondary flow	186	17.9	41	4.4
[7]	Hydrophoretic	2.6	10000	>10000	3
[8]	Hydrodynamic Filtration	200	46.6	124	7.3
OUR WORK	Vertical sheath, hydrodynamics	28000	13	>10000	5

Supplementary Table S2: WBCs sorting comparison

Reference	*Mechanism*	*Throughput 10^8 *cells /min*	*Recovery*	*Enrichment*
[4]	Hydrodynamic filtration with no inertia	0.1	80	3329.33
[9]	Deterministic Margination with no inertia	7.5	80	50
[7]	Hydrophoretic	0.00024	85	210
[10]	Filter with back flush	1.87	72	148
[11]	Magnetic	0.000415	97.4	
[12]	DEP	0.0085	92.1	7
[13]	Cross flow filtration	0.835	27.4	
[6]	Inertial separation	0.019	89.7	
[14]	Inertial separation	0.5	98.4	20
[15]	DLD	0.415	99	
[16]	Hydrodynamic filtration	0.1		29
[17]	Margination based separation	0.015	94	46
OUR WORK	Vertical sheath, hydrodynamics	0.1	73	87

Supplementary Table S3: Estimated H_f for different studies

Study	*Sample Flow rate (ml/min)*	*Vertical Sheath Flow rate (ml/min)*	*Horizontal Sheath Flow rate (ml/min)*	*Qi/Qs*	*H_f (Calculated from equation (7))µm*
Width Optimization	0.02	0.04	0.05	2/4	6.5
Angle Optimization	0.05	0.07	0.05	5/7	3.54
Flow rate Study	0.02	0.04	0.05	2 /4	6.5
	0.05	0.07	0.05	5/7	8.125
	0.05	0.055	0.05	5/5.5	9.285
	0.05	0.09	0.05	5/9	6.9
Vertical sheath study	0.02	0.04	0.05	2/4	6.5
Resolution Study	0.05	0.09	0.05	5/9	7.7

REFERENCES

1 Rhee, M. *et al.* Synthesis of Size-Tunable Polymeric Nanoparticles Enabled by 3D Hydrodynamic Flow Focusing in Single-Layer Microchannels. *Advanced Materials* **23**, H79-H83 (2011).

2 Lee, G.-B., Chang, C.-C., Huang, S.-B. & Yang, R.-J. The hydrodynamic focusing effect inside rectangular microchannels. *Journal of Micromechanics and Microengineering* **16**, 1024 (2006).

3 Stiles, T. *et al.* Hydrodynamic focusing for vacuum-pumped microfluidics. *Microfluidics and Nanofluidics* **1**, 280-283 (2005).

4 Wang, X., Liedert, C., Liedert, R. & Papautsky, I. A disposable, roll-to-roll hot-embossed inertial microfluidic device for size-based sorting of microbeads and cells. *Lab on a Chip* **16**, 1821-1830 (2016).

5 Yamada, M., Seko, W., Yanai, T., Ninomiya, K. & Seki, M. Slanted, asymmetric microfluidic lattices as size-selective sieves for continuous particle/cell sorting. *Lab on a Chip* (2017).

6 Wu, Z., Chen, Y., Wang, M. & Chung, A. J. Continuous inertial microparticle and blood cell separation in straight channels with local microstructures. *Lab Chip* **16**, 532-542 (2016).

7 Choi, S., Song, S., Choi, C. & Park, J.-K. Continuous blood cell separation by hydrophoretic filtration. *Lab on a Chip* **7**, 1532-1538 (2007).

8 Chiu, Y.-Y., Huang, C.-K. & Lu, Y.-W. Enhancement of microfluidic particle separation using cross-flow filters with hydrodynamic focusing. *Biomicrofluidics* **10**, 011906 (2016).

9 Kim, B., Choi, Y. J., Seo, H., Shin, E. C. & Choi, S. Deterministic Migration-Based Separation of White Blood Cells. *Small* **12**, 5159-5168 (2016).

10 Cheng, Y., Ye, X., Ma, Z., Xie, S. & Wang, W. High-throughput and clogging-free microfluidic filtration platform for on-chip cell separation from undiluted whole blood. *Biomicrofluidics* **10**, 014118 (2016).

11 Han, K.-H. & Frazier, A. B. Paramagnetic capture mode magnetophoretic microseparator for high efficiency blood cell separations. *Lab on a Chip* **6**, 265-273 (2006).

12 Han, K.-H. & Frazier, A. B. Lateral-driven continuous dielectrophoretic microseparators for blood cells suspended in a highly conductive medium. *Lab on a Chip* **8**, 1079-1086 (2008).

13 Li, X., Chen, W., Liu, G., Lu, W. & Fu, J. Continuous-flow microfluidic blood cell sorting for unprocessed whole blood using surface-micromachined microfiltration membranes. *Lab on a Chip* **14**, 2565-2575 (2014).

14 Wu, L., Guan, G., Hou, H. W., Bhagat, A. A. S. & Han, J. Separation of leukocytes from blood using spiral channel with trapezoid cross-section. *Analytical chemistry* **84**, 9324-9331 (2012).

15 Choi, J., Hyun, J.-c. & Yang, S. On-chip extraction of intracellular molecules in white blood cells from whole blood. *Scientific reports* **5** (2015).

16 Yamada, M. & Seki, M. Hydrodynamic filtration for on-chip particle concentration and classification utilizing microfluidics. *Lab on a Chip* **5**, 1233-1239 (2005).

17 Jain, A. & Munn, L. L. Biomimetic postcapillary expansions for enhancing rare blood cell separation on a microfluidic chip. *Lab on a chip* **11**, 2941-2947 (2011).
